# Evaluation of the Stress Tolerance of *Salmonella* with Different Antibiotic Resistance Profiles

**DOI:** 10.1155/2021/5604458

**Published:** 2021-09-14

**Authors:** Xingning Xiao, Biao Tang, Siyi Liu, Yujuan Suo, Hua Yang, Wen Wang

**Affiliations:** ^1^State Key Laboratory for Managing Biotic and Chemical Threats to the Quality and Safety of Agro-Products, MOA Laboratory of Quality & Safety Risk Assessment for Agro-Products (Hangzhou), Institute of Agro-Product Safety and Nutrition, Zhejiang Academy of Agricultural Sciences, Hangzhou 310021, China; ^2^College of Food Science and Engineering, Hainan University, Haikou 570228, China; ^3^Institute for Agri-Food Standards and Testing Technology, Shanghai Academy of Agricultural Science, Shanghai 201403, China

## Abstract

Disease caused by antibiotic-resistant *Salmonella* is a serious clinical problem that poses a great threat to public health. The present study is aimed at assessing differences in bacterial kinetics with different antibiotic resistance profiles under environmental stress and at developing microbial tolerance models in lettuce during storage from 4 to 36°C. The drug-resistance phenotypes of 10 *Salmonella* Typhimurium (*S*. Typhimurium) isolates were examined using the broth microdilution method. The results of 10 S. Typhimurium isolates in the suspensions showed that a slow trend towards reduction of drug-sensitive (DS) isolates in relation to the others though without statistical difference. Compared to DS *S*. Typhimurium SA62, greater bacterial reduction was observed in multidrug-resistant (MDR) *S*. Typhimurium HZC3 during lettuce storage at 4°C (*P* < 0.05). It was likely that a cross-response between antibiotic resistance and food-associated stress tolerance. The greater growth in lettuce at 12°C was observed for DS *S*. Typhimurium SA62 compared to MDR *S*. Typhimurium HZC3 and was even statistically different (*P* < 0.05), while no significant difference was observed for bacterial growth between MDR *S*. Typhimurium HZC3 and DS *S*. Typhimurium SA62 strains in lettuce storage from 16 to 36°C (*P* > 0.05). The goodness-of-fit indices indicated the Log-linear primary model provided a satisfactory fit to describe the MDR *S*. Typhimurium HZC3 and DS *S*. Typhimurium SA62 survival at 4°C. A square root secondary model could be used to describe the effect of temperature (12, 16, 28, and 36°C) on the growth rates of *S*. Typhimurium HZC3 (*adj* − *R*^2^ = 0.91, RMSE = 0.06) and *S*. Typhimurium SA62 (*adj* − *R*^2^ = 0.99, RMSE = 0.01) derived from the Huang primary model. It was necessary to pay attention to the tolerance of antibiotic resistant bacteria under environmental stress, and the generated models could provide parts of the input data for microbial risk assessment of *Salmonella* with different antibiotic resistance profile in lettuce.

## 1. Introduction

*Salmonella* is a zoonotic bacterium and is among the most important pathogens causing bacterial foodborne diseases. About 70-80% of foodborne disease outbreaks are caused by *Salmonella* in China [1]. In 2016, a total of two *Salmonella* outbreaks in Australia have been attributed to fresh produce and *Salmonella* Anatum outbreak (144 cases) linked to prepackage lettuce and *Salmonella* Hvittingfoss (97 cases) linked to rock melons [2]. S*almonella* contamination occurs during both production and preparation phases by exposure to contaminated water, soil, insect, or animal fecal matter and through cross-contamination [3]. Animal manure used as fertilizers is a potential source of contamination of raw vegetables [4]. Consumers have also been known to prepare ready-to-eat salad fruits and vegetables with utensils previously used to prepare raw chicken for cooking. This has led to cross-contamination, growth, and exposure to and illness from *Salmonella* of chicken origin [5]. A systematic review of *Salmonella* contamination of lettuce indicated that *Salmonella* prevalence in lettuce was 2.1, 1.0, and 16.9% for farm, industry, and retail markets, respectively [3]. These pose a serious threat to the public health.

Over the past decades, emerging antibiotic resistance has become a global concern, and although procedures have been adopted to avoid resistance spread, antibiotic-resistant bacterial strains among food isolates are still observed [6]. Antibiotic-resistant bacteria, especially zoonotic bacteria such as *Salmonella* which infect edible animals, can be transmitted to humans *via* the food chain or through skin contact [7]. *Salmonella* isolates displaying resistance to clinically important antibiotic agents have increased in China and other countries [4, 8]. A previous study collected 406 raw vegetable samples from retail markets in 39 Chinese cities, and the prevalence of *Salmonella* in lettuce, coriander, cucumber, and tomato were 6.0, 7.8, 0.8, and 1.0%, respectively. In total, 4 (26.7%) *Salmonella* isolates with multidrug-resistant (MDR) phenotypes were detected in raw vegetable samples, including two isolates from lettuce and two isolates from coriander [4]. *Salmonella* isolates in lettuce displayed higher frequency of MDR phenotypes than other types of raw vegetables [4, 9]. Pathogenic bacteria in foods usually experience many stress conditions during food production and processing, which could lead the bacteria into the sublethal injury and viable but nonculturable state. Such conditions can be chlorine or acid chemical treatments or physical stresses, such as cold or heat treatments [10]. Drug-resistant (DR) *Staphylococcus aureus* (*S. aureus*) has been found to be more resistant to acid, heat, and osmotic pressure than drug-sensitive (DS) bacteria [10]. The DR *Escherichia coli* O157:H7 (*E. coli* O157:H7) was sensitive to cold stress during yogurt and juice storage at 4°C [11]. Besides, no significant difference for the tolerance of DS and DR *Listeria monocytogenes* (*L. monocytogenes*) to thermal stress was reported [12]. It was necessary to pay attention to the tolerance of antibiotic-resistant bacteria under environmental stress. Concerning growth and survival kinetics of *Salmonella* with different antibiotic resistance profiles in the suspensions and lettuce, research in this area was still scarce.

Predictive microbiology has been used as an important tool to improve food safety by developing mathematical models to quantitatively predict the growth or survival of microorganisms under prescribed environmental conditions during food processing [13, 14]. Currently, there exists a wealth of data and models that can be used to predict the changes in the *Salmonella* in lettuce during storage [15, 16]. For example, *Salmonella* spp. were inoculated separately in lettuce and stored at 5 to 37°C, and growth curves were constructed by fitting the data to the Baranyi model [16]. A chicken isolate of *Salmonella* Newport (*S.* Newport) has also been used to construct an artificial neural network model in lettuce during storage at temperature of 16 to 40°C as well as *Salmonella* Enterica (*S.* Enterica) ATCC 13076 in minimally processed lettuce as a function of temperature [9, 15]. There was evidence that the growth and survival patterns of DR strains were different from their DS counterparts during treatment at different temperatures, while few model that included the growth and survival of *Salmonella* with different antibiotic resistance in lettuce [17]. There is a need to incorporate into models on the variability of microbial responses with different antibiotic resistance, which will improve the utility of predictive models for exposure assessment and decrease the variability of the quantitative microbial risk assessment (QMRA) model [17, 18].

The objectives of the current study were to (i) assess differences of growth and survival kinetics of isolated strains with different antibiotic resistance profiles to environmental stress and to (ii) construct mathematical models to describe the behaviors of *S*. Typhimurium with different antibiotic resistance profiles in lettuce storage from 4 to 36°C.

## 2. Material and Methods

### 2.1. Bacterial Strains and Culture Conditions

Ten strains of *S.* Typhimurium (SA62, Z62, Z95, Z9, X79, Z27, HZC3, X54, X100 and X63) isolated from anal swabs in poultry farm in Guangdong province were used in the study. These isolates were stored in brain heart infusion broth (BHI, Becton Dickinson (BD), Franklin Lakes, NJ, USA) containing 20% glycerol at -80°C. Each strain was separately incubated in BHI at 37°C for 24 h and cultured to approximately 9 log CFU/mL. Appropriate 10-fold dilutions in sterile phosphate buffered saline (PBS, Sigma, St. Louis, MO, USA) were made and plated on Xylose Lysine Tergitol-4 (XLT4, BD, Franklin Lakes, NJ, USA) agar incubated 37°C for 18 h to determine number of colonies forming units per milliliter in the suspensions.

### 2.2. Antibiotic Susceptibility Tests

Bacterial suspensions were prepared by suspending 3-5 individual colonies grown at 37°C for 18 h on Trypticase Soy agar (TSA, BD, Franklin Lakes, NJ, USA) into 3 mL of 0.9% saline, equivalent to the turbidity of a 0.5 McFarland standard. The 0.5 McFarland inoculum suspensions were further diluted at 1 : 100 in Mueller-Hinton broth (MH, BD, Franklin Lakes, NJ, USA). Then, the panel of antibiotic agents was reconstituted by adding 200 *μ*L/well of the inoculum and incubated at 37°C for 18 h. Antibiotic susceptibility testing was performed using the broth microdilution method with the commercial Gram-negative antibiotic panel (Biofosun, Fosun Diagnostics, Shanghai, China) consisting of ampicillin (resistant, AMP ≥ 32 *μ*g/mL), amoxicillin/clavulanate (resistant, AMC ≥ 32 *μ*g/mL), cefotaxime (resistant, CTX ≥ 4 *μ*g/mL), meropenem (resistant, MEM ≥ 4 *μ*g/mL), amikacin (resistant, AMK ≥ 64 *μ*g/mL), gentamicin (resistant, GEN ≥ 16 *μ*g/mL), colistin (resistant, CS ≥ 2 *μ*g/mL), ceftiofur (resistant, CEF ≥ 8 *μ*g/mL), ciprofloxacin (resistant, CIP ≥ 1 *μ*g/mL), sulfamethoxazole (resistant, T/S ≥ 4/76 *μ*g/mL), tetracycline (resistant, TET ≥ 16 *μ*g/mL), tigecycline (resistant, TIG ≥ 8 *μ*g/mL), and florfenicol (resistant, FFC ≥ 16 *μ*g/mL). The breakpoints for each antibiotic agent were set by the Clinical and Laboratory Standards Institute and the European Committee on Antibiotic Susceptibility Testing [19, 20].

### 2.3. Assessment of Salmonella Stress Tolerance

Each isolate was allowed to achieve a final concentration of approximately 9 log CFU/mL, as described in Section 2.1. The initial concentrations of suspensions to the environmental stress were selected based on the preliminary test and previous study [21]. To evaluate the effect of low storage temperatures on the tolerance of *Salmonella*, suspensions containing approximately 3 log CFU/mL were stored at 4, 12, 24, and 36°C. Sampling was carried out at varying time intervals depending on the storage temperature. To assess the heat tolerance of *Salmonella*, a total of 10 mL suspensions containing approximately 8 log CFU/mL were poured into a glass tube and exposed to hot water at 60 and 75°C for 2 min in a laboratory water bath (TX150, Grant, Royston, UK) equipped with a digital thermometer (34970A, Agilent, Santa Clara, CA, USA) to monitor both temperatures of water and bacterial suspensions. Sodium hypochlorite (NaClO) stock solution containing 56.8 mg/mL chlorine (Sangon Biotech Co., Ltd., Shanghai, China) was diluted with sterile Milli-Q water (PALL, Buckinghamshire, UK), and the concentration in solution was 100 and 200 mg/L determined using a Palintest ChlorSense meter (KEMS10DISCN, Gateshead, Tyne and Wear, UK). To examine chlorine tolerance, *Salmonella* suspensions with initial concentration of approximately 5 log CFU/mL were inoculated into NaClO solution of 100 and 200 mg/L for 10 min. In the preliminary tests, XLT4 agar was compared with TSA in plate counting, and only less than 0.1 log CFU/mL difference was observed, which was not significantly different (*P* > 0.05) as determined by ANOVA. Therefore, the *Salmonella* were selectively enumerated on XLT4 agar using a spiral plater (WASP 2, Don Whitley Scientific, Shipley, UK). The plates were incubated at 37°C for 18 h, and colonies were enumerated using a ProtoCOL 3 automated colony counter (Synbiosis, Cambridge, UK). The viable bacterial populations on samples were expressed as CFU/mL, and the detection limit was 1.3 log CFU/mL. Triplicate experiments were performed for each isolate.

### 2.4. Evaluation of Salmonella Stress Tolerance in Lettuce Storage

Lettuce was purchased at supermarket in Hangzhou, China, and these samples were transported to the laboratory using ice bags. Debris and other particles of lettuce leaves were washed with running tap water to reduce natural microbiota load, dried inside a laminar flow safety cabinet, and exposed to UV for 30 min. The intact leaves were cut into 5 × 5 cm pieces using a sterile surgical knife and put it into the disinfected plastic plates. These samples were divided into two groups. One group was processed for inspection process, and no *Salmonella* was initially present on the three pieces of lettuce. The other group was processed for the storage study. The samples were submerged into *Salmonella* suspensions containing approximately 5 log CFU/mL for 10 min and were dried inside a laminar flow safety cabinet for another 30 min. The initial inoculum level on the lettuce was 3 ± 0.2 log CFU/cm^2^. Inoculated portions of lettuce were stored in stomacher bags at 4, 12, 16, 28, and 36°C. Sampling was carried out at varying time intervals depending on the storage temperature. At each time point, three lettuce samples were individually added to sterile stomacher bags (Seward, London, UK) containing 25 mL buffered peptone water (BPW, BD, Franklin Lakes, NJ, USA) and homogenized for 1 min in a Model 400 food stomacher (Seward, London, UK). The homogenates were serially 10-fold diluted in BPW, and a 50 *μ*L portion of appropriate dilutions was plated in duplicate onto the XLT4 agar using a spiral plater. The plates were incubated at 37°C for 16 h. Colonies on XLT4 agar plates were enumerated by a ProtoCOL 3 automated colony counter. The viable bacterial populations in lettuce samples were expressed as CFU/cm^2^, and the detection limit was 1.3 log CFU/cm^2^. Each treatment was repeated twice on different days using duplicate plates for each sample.

### 2.5. Microbial Modeling in Lettuce Storage

The primary model is used to predict the number of bacteria in food at a constant temperature [18]. The growth and survival patterns of *Salmonella* under different storage temperatures were plotted as the log of the population size (log *N*_*t*_) against time (*t*) and were analyzed to develop primary models. For the survival curve of *Salmonella* under 4°C, a Log-linear model was used. This model assumes a homogeneous bacterial resistance to refrigeration described with a linear relationship between the log of the population density and storage time. It has been frequently used for bacterial survival curve fitting [22, 23].

(1) $$\begin{array}{c} \log N_{t}=\log N_{0}-\frac{t}{D}, \end{array}$$

where *N*_*t*_ (CFU/cm^2^) is the bacterial population at time *t*, *N*_0_ is the initial bacterial population (CFU/cm^2^), and *D* is the decimal reduction time (h) at a specific treatment temperature.

Growth curves of *Salmonella* incubated at 12, 16, 28, and 36°C utilized four primary models from (2) to (5) that were chosen to fit the growth data collected from storage experiments. Their performances were compared to determine the model with the best fit.

The Modified Gompertz model (2) has been widely used in bacterial growth modeling studies [22].

(2) $$\begin{array}{c} \log N_{t}=\log N_{0}+\log\frac{N_{\max}}{N_{0}}\times \exp\left\{-\exp\left[\frac{2.718\mu_{\max}}{\log\left(N_{\max}/N_{0}\right)}\times \text{m}\left(\lambda -t\right)+1\right]\right\}. \end{array}$$

The Huang model (3) is especially suitable for growth curves with three phases (lag, exponential, and stationary) [24].

(3) $$\begin{array}{c} \log N_{t}=\log N_{0}+\log N_{\max}-\log\left(\exp\left(\log N_{0}\right)+\left(\exp\left(\log N_{0}\right)-\exp\left(\log N_{0}\right)*\exp\left(-\mu_{\max}*\left(B\right)\right)\right)\right),\\ B=t+0.25*\log\frac{\left(1+\exp\left(-4*\left(t-\lambda \right)\right)\right)}{1+\exp\left(4*\lambda \right)}. \end{array}$$

The logistic model has been widely used to describe the growth curve like the sigmoidal type on (4) [25].

(4) logNt=logN0+logNmax−logN01+tt0^μmax.

The Baranyi model assumes that during the lag phase, the specific growth rate depends on the need of each cell to synthesize an intracellular substance referred to as a bottleneck-modeling function on (5) [26].

(5) $$\begin{array}{c} \log N_{t}=\log N_{0}+\mu_{\max}A\left(t\right)-\ln\left(1+\frac{\exp\left(\mu_{\max}A\left(t\right)\right)-1}{\exp\left(\log N_{\max}-\log N_{0}\right)}\right),\\ A\left(t\right)=t+\frac{1}{\mu_{\max}}\ln\left(\exp\left(-\mu_{\max}t\right)+\exp\left(-h_{0}\right)-\exp\left(-\mu_{\max}t-h_{0}\right)\right). \end{array}$$

In Equations (2)–(5), *N*_0_, *N*_max_, and *N*_*t*_ (CFU/cm^2^) represent the initial, maximum, and time *t* (h) bacterial populations, respectively, *λ* is the lag time (h), *μ*_max_ is the maximum specific growth rate (log CFU/cm^2^/h), and *h*_0_ is the physiological state of the microorganism.

The secondary model was used to predict parameters obtained from the primary model with temperature changes. The square root model (6) has proven its efficiency to describe the temperature dependence of bacterial growth in suboptimal temperatures [27]:

(6) $$\begin{array}{c} \sqrt{\mu_{\max}}=b\left(T-T_{\min}\right). \end{array}$$

In Equation (6), *μ*_max_ is the maximum growth rate at each temperature (*T*), and *b* and *T*_min_ are the model parameters, with *T*_min_ defined as the minimum growth temperature.

### 2.6. Model Evaluation and Validation

ANOVA was used to evaluate significance and adequacy of the model. Fitting goodness of the model was characterized by the correlation coefficient (*adj* − *R*^2^), Akaike information criterion (AIC), and the root mean square error (RMSE). For model validation, bias factors (*B*_*f*_) on (7) and accuracy factors (*A*_*f*_) on (8) were calculated with the data selected from literatures [28–30]. The selected data should be (i) describing the behavior of *Salmonella* were generated in lettuce, (ii) obtained using the traditional plate counts method, and (iii) obtained in the temperature range from 4 to 36°C. Finally, *Salmonella* survival data in lettuce at 4°C were used to validate the Log-linear model, and growth data in lettuce at 15 and 20°C were used to estimate the applicability of *μ*_max_ with the square root model [29, 30].

(7) $$\begin{array}{c} B_{f}=10^{\left[\overset{n}{\underset{i=1}{\sum }}\log\left(M_{\text{obs}}/M_{\text{pred}}\right)/n\right]}, \end{array}$$

(8) $$\begin{array}{c} A_{f}=10^{\left[\overset{n}{\underset{i=1}{\sum }}\left|\text{log}\left(M_{\text{obs}}/M_{\text{pred}}\right)\right|/n\right]}, \end{array}$$

where *n* is the number of trials, *M*_obs_ is the observed specific bacterial reductions at 4°C or specific growth rate at 15 and 20°C, and *M*_pred_ is the predicted bacterial reductions at 4°C or specific growth rate at 15 and 20°C.

### 2.7. Statistical Analysis

The results of bacterial growth and survival on samples were analyzed by calculating the means and standard deviations using Excel 2010 (Microsoft, Redmond, WA, USA). Statistical analyses consisted of ANOVA with Duncan's test, using the SPSS 19.0 software (IBM, Chicago, IL, USA). A significant difference was established at *P* < 0.05.

## 3. Results and Discussion

### 3.1. Phenotypic Antibiotic Resistance

As shown in Table 1, *S*. Typhimurium SA62 and Z62 were susceptible to all 13 antibiotics; *S*. Typhimurium Z95, Z9, X79, and Z27 were resistant to one or two antibiotics; and *S*. Typhimurium HZC3, X54, X100 and X63 were resistant to at least three antibiotics and therefore were considered MDR. Of the 10 *Salmonella* isolates analyzed, six isolates were resistant to FFC, and six isolates were also resistant to TET. A total of 4 *Salmonella* isolates were resistant to both FFC and TET. These results were consistent with the reported rates from previous study of resistance among *Salmonella* isolates from other food sources in China [4]. FFC is an antibiotic that is exclusively used in veterinary medicine in China, and TET is commonly used for the treatment of infections in poultry, which could be an explanation for the high rates of resistance towards these antibiotics [31].

### 3.2. Growth and Survival of Salmonella through Environmental Stress

**Figure 1:**
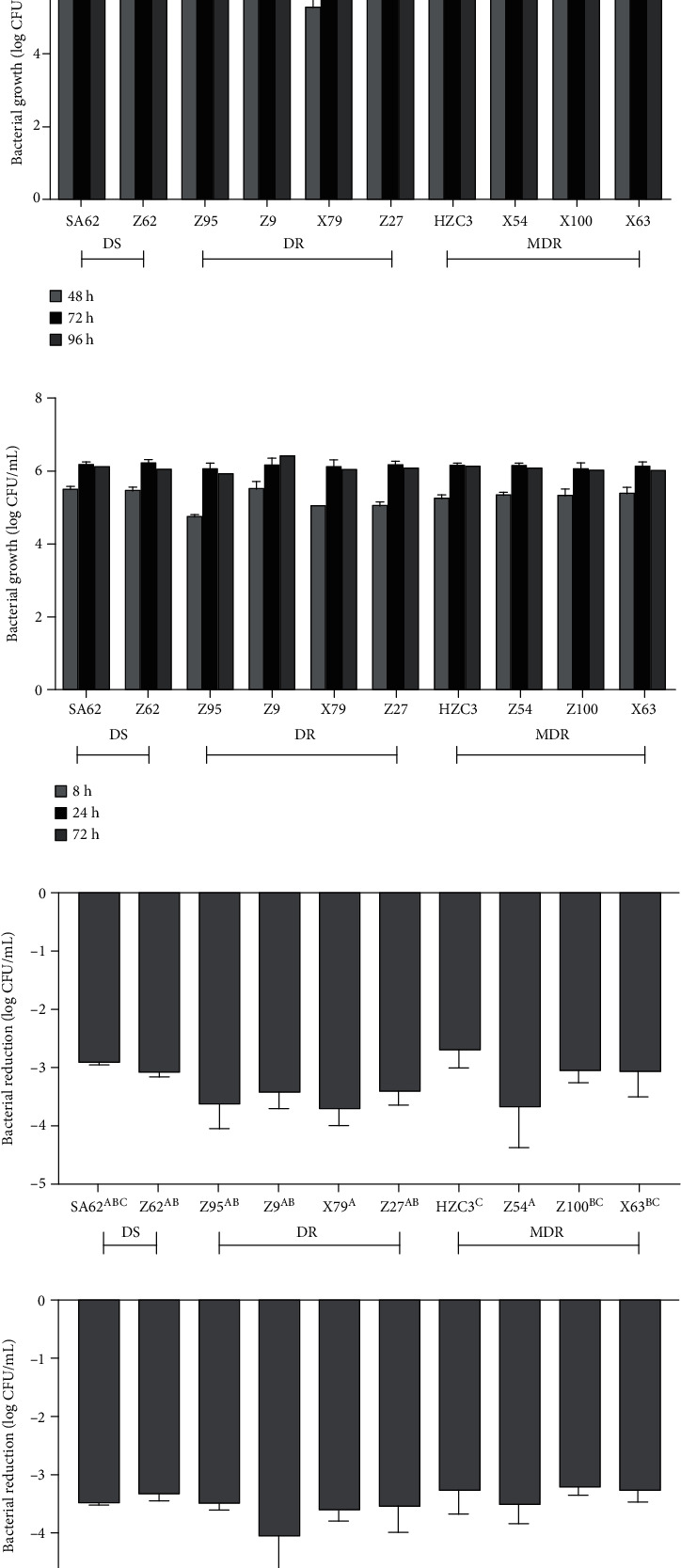
Logarithmic reduction or growth of 10 isolates of *S*. Typhimurium in the suspensions after exposure to storage temperatures at (a) 4°C, (b) 12°C, (c) 24°C, and (d) 36°C for 96 h; hot water temperatures at (e) 60°C and (f) 75°C for 2 min; NaClO concentrations at (g) 100 mg/L and (h) 200 mg/L for 10 min. DS: drug-sensitive; DR: drug-resistant; MDR: multidrug-resistant. Uppercase letters represent the results of significant difference analysis with different isolates. Where an uppercase letter is not shown, no statistical difference between all the data is analyzed.

Logarithmic reduction and growth of 10 isolates of *Salmonella* in the suspensions after exposure to different storage temperatures, hot water temperatures and NaClO concentrations are plotted in Figure 1. The results showed that the ranges of reductions in DS, DR, and MDR *Salmonella* strains after storage at 4°C for 96 h were 0.02-0.07, 0.03-0.16, and 0.02-0.30 log CFU/mL, respectively (Figure 1(a)). There was a slow trend towards reduction of DS isolates in relation to the others but without statistical difference. No significant differences in bacterial growth with different antibiotic profiles were observed during storage at 12, 24, and 36°C (*P* > 0.05) (Figures 1(b)–1(d)). As shown in Figures 1(e) and 1(f), the reductions of *Salmonella* isolates in the suspensions at the end of 2 min treatments were 3.28 ± 0.35 and 3.50 ± 0.24 log CFU/mL at 60 and 75°C, respectively. The reductions of *Salmonella* isolates in the suspensions were 0.25 ± 0.10 and 0.32 ± 0.09 log CFU/mL with 100 and 200 mg/L of chlorine, respectively (Figures 1(g) and 1(h)). After heat and chlorine exposure, lower trends towards mortality of DS strains compared with DR or MDR strains were found though without statistical difference (Figures 1(e)–1(h)).

**Figure 2:**
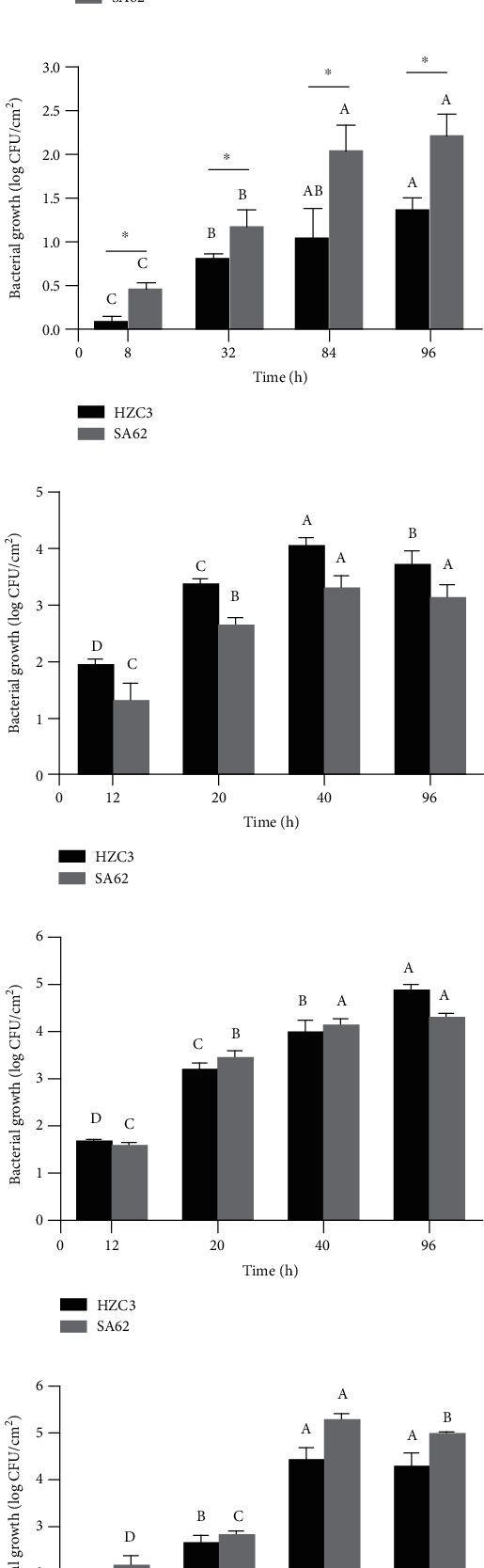
Bacterial survival and growth of MDR *S*. Typhimurium HZC3 and DS *S*. Typhimurium SA62 in lettuce during storage at (a) 4°C, (b) 12°C, (c) 16°C, (d) 28°C, and (e) 36°C. The asterisk (^∗^) represents significant differences between the two isolates at the same incubation time and temperature. Uppercase letters represent significant differences for the same isolate at the same temperature during the incubation times.

In the test of bacterial storage in lettuce, *S*. Typhimurium HZC3 and SA62 were selected to investigate the difference of bacterial kinetics with different antibiotic resistance profiles during storage. The criteria used to choose these two isolates should be (i) MDR and DS strains, and (ii) there was a larger difference in bacterial reduction between MDR and DS strains to the environmental stress. In Figure 2(a), the bacterial reductions for the MDR *S*. Typhimurium HZC3 and DS *S*. Typhimurium SA62 in lettuce at the end of 120 h storage were 1.36 ± 0.21 and 0.84 ± 0.04 log CFU/cm^2^ at 4°C, respectively. Greater bacterial reductions were observed for MDR *S*. Typhimurium HZC3 at 4°C from 56 to 108 h (*P* < 0.05). The greater growth in lettuce at 12°C was observed for DS *S*. Typhimurium SA62 compared to MDR *S*. Typhimurium HZC3 and was even statistically different (*P* < 0.05) (Figure 2(b)), while there was no significant difference of bacterial growth between DS *S*. Typhimurium SA62 and MDR *S*. Typhimurium HZC3 in lettuce storage from 16 to 36°C (*P* > 0.05) (Figures 2(c)–2(e)). Overall, bacterial reductions in the suspensions were lower than that in lettuce. The reason could be that the bacterial on the lettuce surface immediately in contact with cold may be killed. In the study of Food Standards Agency funded data generated at Institute of Food Research (https://browser.combase.cc/), reduction of *Salmonella* in broth was 0.05 log CFU/mL after 72 h storage at 4°C, which was lower than that in the iceberg lettuce (~0.68 log CFU/g) [29].

In this study, there was a rapid trend towards reduction of MDR isolates to cold stress in broth, and significant greater bacterial reductions were observed for MDR *S*. Typhimurium HZC3 in lettuce. No significant differences for the tolerance of antibiotic susceptible and resistant *Salmonella* were found to thermal and chlorine pressure in the broth. The effects of antibiotic resistance profiles on cold, thermal, and chlorine tolerance of bacteria have been reported in many studies [11, 12, 32]. The observations have been found that the DR *E. coli* O157:H7 died off significantly faster than the DS strains in both yogurt and juice at 4°C (*P* < 0.05) [11]. A previous study found no significant differences for the tolerance of antibiotic susceptible and resistant *L. monocytogenes* to thermal stress at 58°C [12]. As well, the chlorine stress of acidified sodium chloride resulted in no difference of the inactivation level for antibiotic resistant and susceptible *Salmonella* [32]. Other studies have shown that DR *L. monocytogenes* and *Salmonella* were more tolerant than DS strains when exposed to environmental stressors [10, 12]. Sigma factors are the well-known general regulators of bacteria in response to cold and heat stresses [17, 33]. For the chlorine stress, the treatment of *Salmonella* Enteritidis would induce the overexpression of marRAB operon, a global antibiotic resistance regulator which involved in the production of AcrAB efflux pumps to extrude antibiotics [34]. A study found that the inhibition of efflux pumps with reserpine and thioriodazine in antibiotic resistant *L. monocytogenes* decreased the MIC of disinfectants-hydrogen peroxide and benzalkonium chloride by two to eight folds [12].

### 3.3. Parameter Estimates of the Microbial Models in Lettuce

**Figure 3:**
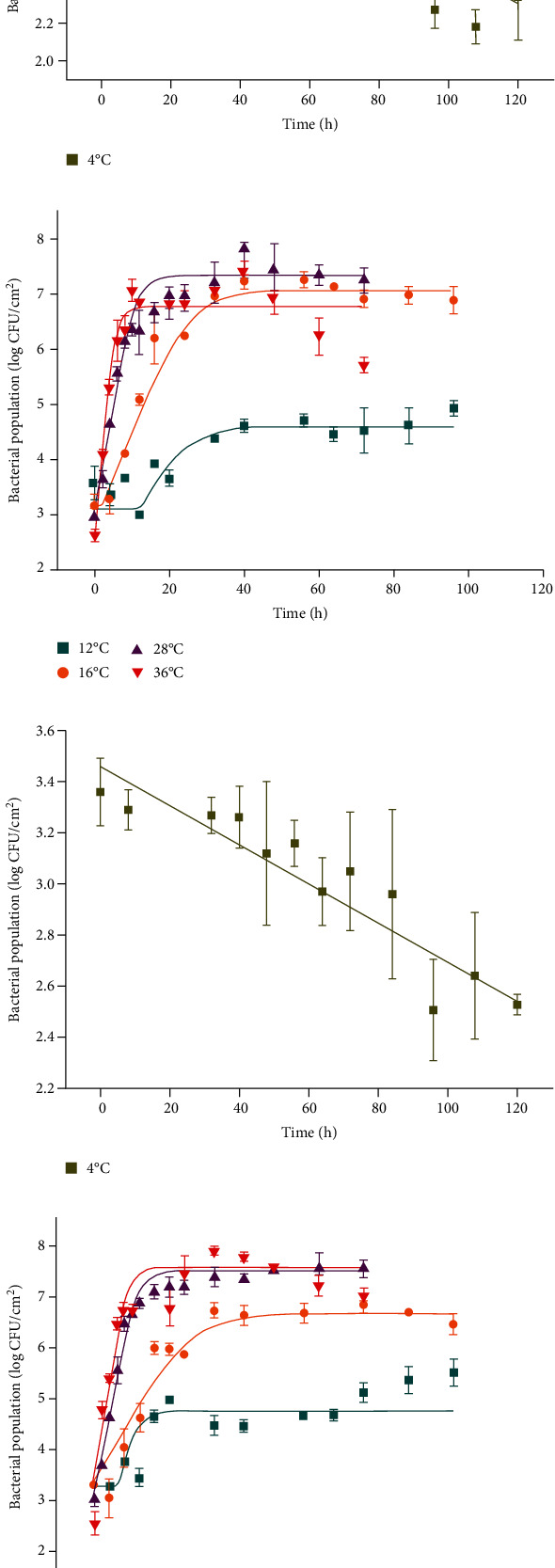
Log-linear and Huang models fitting the survival and growth of MDR *S*. Typhimurium HZC3 and DS *S*. Typhimurium SA62 in lettuce storage: (a) HZC3 at 4°C; (b) HZC3 at 12, 16, 28, and 36°C; (c) SA62 at 4°C; (d) SA62 at 12, 16, 28, and 36°C.

Significant differences in bacterial reductions were found for MDR *S*. Typhimurium HZC3 and DS *S*. Typhimurium SA62 strains that were related to storage time at 4°C (*P* < 0.05). The target *adj* − *R*^2^ (> 0.9) and RMSE (< 0.2) values were reached suggesting that the Log-linear model was reasonably accurate in describing these effects (Figure 3 and Table 2). The Log-linear model assumes a homogeneous bacterial die-off rate, and therefore, a linear curve was applied. A mechanistic explanation for this was that bacterial death was the result of inactivation of some critical enzyme or enzyme system and governed by first-order inactivation kinetics [35].

Significant differences in *Salmonella* growth in lettuce were found at 12, 16, 28, and 36°C over time (*P* < 0.05). As the incubation temperature was increased, the lag phases of *Salmonella* were shortened while the growth rates increased [36]. The Modified Gompertz, Huang, Logistic, and Baranyi models were used to fit both MDR *S*. Typhimurium HZC3 and DS *S*. Typhimurium SA62 growth at 12, 16, 28, and 36°C. The results of the parameter estimates and statistical analysis indicated that the Huang models agreed reasonably well with the observed data (Table 3). The Huang model was successfully used to describe the growth kinetics of *L. monocytogenes* with and without cold-adaption, on fresh-cut cantaloupe under different storage temperatures [37]. Besides, Huang primary model is proper than other models for describing the growth of *S. aureus* in ready-to-eat cooked rice with pork floss [38]. The Huang model is based on the fundamental observation of the classical bacterial growth process that exhibits the three different phases of growth: lag, exponential, and stationary as well as adaption in the lag phase. This model utilizes a transition function to simulate bacterial adaption and to define the lag phase of a growth curve. This allows the model to transit smoothly from the lag phase to the exponential phase [38, 39].

**Figure 4:**
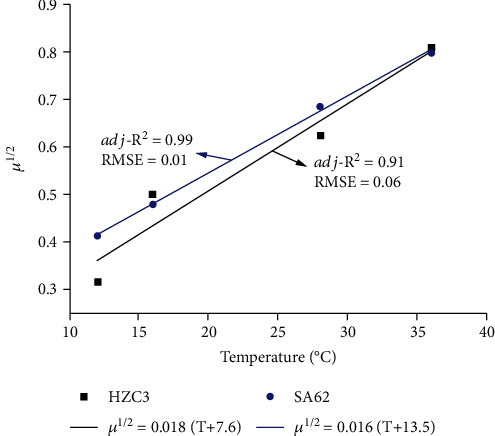
Square root model of the maximum growth rates of MDR *S*. Typhimurium HZC3 and DS *S*. Typhimurium SA62 in lettuce at different storage temperatures. *μ* values were obtained from the Huang model at each temperature.

A square root model was developed to describe the effect of temperature on the growth rates of *S*. Typhimurium HZC3 and SA62 derived from the Huang model. The relationships between the growth rates and temperature for both *S*. Typhimurium HZC3 and SA62 strains followed a linear trend (Figure 4). The model for *S*. Typhimurium HZC3 and *S*. Typhimurium SA62 was in acceptable agreement between predictions and observations (Figure 4). Similar to our findings, a previous study concluded that the square root model could adequately describe the individual effects of temperature (7 ~ 30°C) on the growth rate of *S.* E*nterica* and *L. monocytogenes* in lettuce [15].

### 3.4. Model Validation and Its Application in QMRA

The values of *A*_*f*_ and *B*_*f*_ for the developed models are shown in Table 4. For the bacterial survival model at 4°C, the values of *A*_*f*_ and *B*_*f*_ for the MDR *S*. Typhimurium HZC3 and DS *S*. Typhimurium SA62 ranged from 1.85 to 1.87, and for the bacterial growth at 12 to 36°C, the *A*_*f*_ and *B*_*f*_ values of the square root model for the *S*. Typhimurium HZC3 and SA62 strains ranged from 1.15 to 1.39, indicating the developed models showed a satisfactory performance to predict the bacterial populations. The *A*_*f*_ takes the average distance between every point and the line of equivalence as a measure of how close; on average, the predictions are to the observations [40, 41]. Satisfactory *B*_*f*_ limits are more difficult to define because limits of acceptability are related to the specific application of the model. Ideally, predictive models would have *A*_*f*_ = *B*_*f*_ = 1. When compared to independently published data, *B*_*f*_ values in the range 0.6-3.99 were acceptable for the growth rates of pathogens and spoilage organisms [40]. The *A*_*f*_ value is indicating that 85-87% of the data is over- or underestimated. The validation data were selected from Combase Database (https://browser.combase.cc/). Only one literature study was available on *S.* Typhimurium 14028 at 4°C in iceberg lettuce from Combase Database, which was used to validate the model. More research data on bacterial survival and growth with different antibiotic resistance in lettuce is needed in the future.

Instead of considering all hazard strains as equally likely to cause disease, this study could improve hazard identification by focusing on those strains with different antibiotic resistance profiles and could be used to stratify hazards into strains that are expected to behave difference, e.g., in terms of growth, survival, or response to antibiotic resistance [42]. The QMRA input parameters can be tailored to each strain accordingly, making it possible to capture the variability in the strains of interest while decreasing the uncertainty in the model [43].

## 4. Conclusions

In this study, there was a slow trend towards reduction of DS isolates in relation to the others to the cold, heat, and chlorine stress but without significant difference. In broth, there was a rapid trend towards reduction of MDR isolates to cold stress, and significant greater bacterial reductions were observed for MDR S. Typhimurium HZC3 in lettuce Log-linear models were also able to describe bacterial reductions in lettuce during storage at 4°C. The Huang model was the best fit model to describe bacterial growth at 12, 16, 28, and 36°C, and a square root model was used to describe the effect of temperature on the parameters of growth rates. The generated models could provide parts of the input data for microbial risk assessment of *Salmonella* with different antibiotic resistance profile in lettuce and make it possible to capture the variability in the strains of interest while decreasing the uncertainty in some model input parameters.

## Figures and Tables

**Table 1:** Antibiotic resistance phenotype of *Salmonella* isolates.

Isolation	Phenotypic antibiotic resistance	Number of resistant antibiotics	Classification
SA62	—	0	DS^a^
Z62	—	0	

Z95	TET	1	DR^b^
Z9	CIP--FFC	2	
X79	AMC--FFC	2	
Z27	CIP--TET	2	

X63	CS--T/S--TET--FFC	4	MDR^c^
HZC3	AMP--AMC--CIP--TET--FFC	5	
X54	AMP--MEM--GEN--CEF--T/S--TET--FFC	7	
X100	AMP--AMC--CTX--GEN--CIP--T/S--TET--FFC	8	

^a^DS: bacterial sensitive to all 13 antibiotics, including ampicillin (AMP), amoxicillin/clavulanate (AMC), cefotaxime (CTX), meropenem (MEM), amikacin (AMK), gentamicin (GEN), colistin (CS), ceftiofur (CEF), ciprofloxacin (CIP), sulfamethoxazole (T/S), tetracycline (TET), tigecycline (TIG), and florfenicol (FFC), was DS bacteria. ^b^DR: bacteria resistant to one or two antibiotics were called DR bacteria. ^c^MDR: multidrug resistance was defined as resistance to at least three antibiotics.

**Table 2:** Parameter estimates and statistical analysis of the Log-linear model fitted to MDR *S*. Typhimurium HZC3 and DS *S*. Typhimurium SA62 survival curves at 4°C.

Strain	log *N*_0_	*D*	*adj* − *R*^2^	AIC	RMSE
HZC3	3.48	102.84	0.92	-38.03	0.15
SA62	3.46	131.27	0.95	-35.84	0.17

log *N*_0_: the initial bacterial population in lettuce (CFU/cm^2^); *D*: the decimal reduction time (h) at a specific treatment temperature; *adj* − *R*^2^: the correlation coefficient; AIC: Akaike information criterion; RMSE: the root mean square error.

**Table 3:** Parameter estimates and statistical analysis of the Modified Gompertz, Huang, Logistic, and Baranyi models fitted to MDR *S*. Typhimurium HZC3 and DS *S*. Typhimurium SA62 growth curves at 12, 16, 28, and 36°C.

Model	Strain	Temperature (°C)	log *N*_0_ (CFU/cm^2^)	*λ*/*h*_0_ (h)	*μ*_max_ (log CFU/cm^2^/h)	log *N*_max_ (CFU/cm^2^)	*adj* − *R*^2^	AIC	RMSE
Modified Gompertz	HZC3	12	3.43	14.27	0.07	4.65	0.82	-22.19	0.26
		16	3.08	4.12	0.31	7.01	0.98	-30.17	0.22
		28	2.93	0.00	0.47	7.22	0.96	-34.54	0.29
		36	2.61	0.10	0.83	7.00	0.97	-39.07	0.24
	SA62	12	3.30	11.81	0.47	4.90	0.70	19.40	0.39
		16	3.14	4.71	0.27	6.62	0.96	31.21	0.27
		28	2.63	0.00	0.57	7.36	0.99	-47.47	0.14
		36	2.56	0.00	0.47	7.52	0.89	36.21	0.34

Huang	HZC3	12	3.41	12.32	0.10	4.66	0.84	-23.10	0.25
		16	3.15	3.66	0.25	7.00	0.97	-28.15	0.24
		28	3.04	0.00	0.39	7.19	0.95	-26.16	0.33
		36	2.61	0.00	0.69	6.98	0.97	-36.24	0.24
	SA62	12	3.28	6.11	0.18	4.94	0.88	20.21	0.43
		16	3.19	4.21	0.23	6.63	0.96	31.20	0.27
		28	3.01	0.37	0.47	7.33	0.99	-41.86	0.17
		36	2.56	0.00	0.76	7.31	0.92	-17.38	0.42

Logistic	HZC3	12	3.43	24.63	0.21	4.66	0.82	-31.81	0.26
		16	3.14	11.64	0.25	7.05	0.98	-35.86	0.21
		28	2.89	5.25	0.63	7.48	0.98	-39.12	0.21
		36	2.62	3.11	0.55	7.18	0.98	-38.57	0.23
	SA62	12	3.30	34.06	0.17	5.93	0.67	-20.35	0.41
		16	2.87	11.47	0.37	6.70	0.95	-32.94	0.26
		28	3.28	5.39	0.43	7.45	0.99	-61.42	0.11
		36	3.30	1.17	0.90	7.91	0.91	-31.43	0.30

Baranyi	HZC3	12	3.56	-0.91	0.10	4.72	0.76	-21.95	0.30
		16	3.14	-1.62	0.32	6.97	0.97	-26.34	0.24
		28	3.07	13.74	0.39	7.19	0.95	-25.29	0.33
		36	2.61	67.54	0.69	6.98	0.97	-9.28	0.24
	SA62	12	3.14	0.00	0.18	4.94	0.64	19.91	0.43
		16	2.22	4.58	0.23	6.62	0.95	31.18	0.28
		28	2.84	6.14	0.47	7.33	0.98	-41.30	0.18
		36	4.45	0.00	0.44	7.46	0.86	32.34	0.38

*λ*: the lag time (h); *h*_0_: the physiological state of the microorganism; *μ*_max_: the maximum specific growth rate (log CFU/cm^2^/h); log *N*_max_: maximum bacterial populations (CFU/cm^2^/h).

**Table 4:** *A*_*f*_ and *B*_*f*_ values used for the predictive models of MDR *S*. Typhimurium HZC3 and DS *S*. Typhimurium SA62.

Temperature (°C)	Model	Strain	*A*_*f*_	*B*_*f*_
4	Log-linear	HZC3	1.85	1.85
		SA62	1.87	1.87

12, 16, 28, 36	Square root	HZC3	1.39	1.39
		SA62	1.15	1.15

*A*_*f*_: accuracy factor; *B*_*f*_: bias factor.

## Data Availability

The data used to support the findings of this study are included within the article.
